# Cerebellar heterotopia of infancy in sudden infant death syndrome: an observational neuropathological study of four cases

**DOI:** 10.1007/s00414-020-02316-x

**Published:** 2020-05-21

**Authors:** Jakob Matschke, Jan-Peter Sperhake, Nadine Wilke, Klaus Püschel, Markus Glatzel

**Affiliations:** 1 Forensic Neuropathology Unit, University Medical Centre Hamburg-Eppendorf, Martinistrasse 52, 20246 Hamburg, Germany; 2 Institute of Neuropathology, University Medical Centre Hamburg-Eppendorf, Martinistrasse 52, 20246 Hamburg, Germany; 3 Institute of Legal Medicine, University Medical Centre Hamburg-Eppendorf, Martinistrasse 52, 20246 Hamburg, Germany; 4 Institute of Legal Medicine, University Hospital Schleswig-Holstein, Campus Lübeck, Ratzeburger Allee 160, 23538 Lübeck, Germany

**Keywords:** SIDS, Forensic neuropathology, Malformations of the central nervous system, Arousal circuits

## Abstract

Sudden infant death syndrome (SIDS) is the sudden unexpected death of an infant < 1 year of age that remains unexplained after comprehensive workup including complete autopsy and investigation of the circumstances of death. The triple risk hypothesis posits that SIDS results as a combination of both intrinsic and extrinsic factors on the background of a predisposing vulnerability. Neuropathological examination in the past has focussed mainly on the brainstem as the major player in respiratory control, where subtle findings have been linked to the chain of events leading to death in SIDS. The cerebellum has received less attention, probably due to an assumed negligible role in central cardiorespiratory control. We report four cases of SIDS in which neuropathological investigation revealed cerebellar heterotopia of infancy, a distinct malformation of the cerebellum, and discuss the potential impact of this condition on the aetiology and pathogenesis of SIDS.

## Introduction

Sudden infant death syndrome (SIDS) is defined as any sudden unexpected death of an infant < 1 year of age “with onset of the fatal episode apparently occurring during sleep, that remains unexplained after a thorough investigation, including performance of a complete autopsy and review of the circumstances of death and the clinical history” [1–3].

According to the classical triple risk model, SIDS occurs when an infant is exposed to the simultaneous occurrence of (a) an intrinsic, predisposing vulnerability, (b) during a critical developmental period, and (c) an additional extrinsic factor, e.g., sleeping in the prone position [4]. Due to a failure of physiological arousal and/or autoresuscitation, the endangered infant falls into progressive asphyxia resulting in hypoxic coma and, eventually, death [5]. It is widely believed that many SIDS cases harbour defects in brainstem-mediated protective responses to possible life-threatening events during sleep [6]. Accordingly, various morphological and biochemical abnormalities have been described in the brainstem of SIDS victims, particularly concerning serotonergic transmitters [6, 7].

Conversely, the cerebellum has received much less attention, probably due to an obvious or assumed negligible role in central cardiorespiratory control. Studies on the role of cerebellar morphology on the pathogenesis of SIDS have so far been inconclusive, largely due to different methodological approaches [8–17].

We report four cases of SIDS with findings consistent with cerebellar heterotopia in infancy (CHOI), a distinct developmental anomaly of the cerebellum, and discuss its potential impact on the cause of death. Considering recent advances in our understanding of the physiology of cerebellum, its role in the chain of events leading to death in SIDS may have been underestimated and warrants further investigation.

## Case reports

### Case #1

#### History

This 3-month-old male infant was found lifeless lying in supine position in a bed, which he had shared with his mother. Resuscitation measures were without success. He had been the first child of his 20-year-old mother, who had been smoking throughout pregnancy and thereafter. Two months prior to death, a heart murmur was found, and echocardiography detected slight flow velocity acceleration in the ascending aorta and the left pulmonary artery. Since the boy was clinically inconspicuous, and without signs of cardiac insufficiency at echocardiography, a follow-up investigation was scheduled, but the boy died before that. Further investigation of the circumstance and scene of death by law enforcement did not reveal any suspicious findings. A forensic autopsy was performed on behalf of the public prosecution office.

#### Autopsy findings

External examination was unremarkable. Body weight was 4820 g (6th percentile), body length 59 cm (19th percentile), and head circumference 39.5 cm (8th percentile). There were multiple petechial haemorrhages over the thymus and visceral pleura. The myocardium of the left ventricle was thickened (12.0 mm—normal value: 6.4 ± 1.7 mm [18]). Histology revealed hyperplastic bronchus-associated lymphatic tissue and minor pulmonary inflammatory infiltrates, considered non-sufficient to be an unequivocal cause of death. The myocardium showed no microscopic abnormalities. After thorough discussion, the case was classified as SIDS category II according to the San Diego scheme [1].

#### Neuropathology findings

**Fig. 1:**
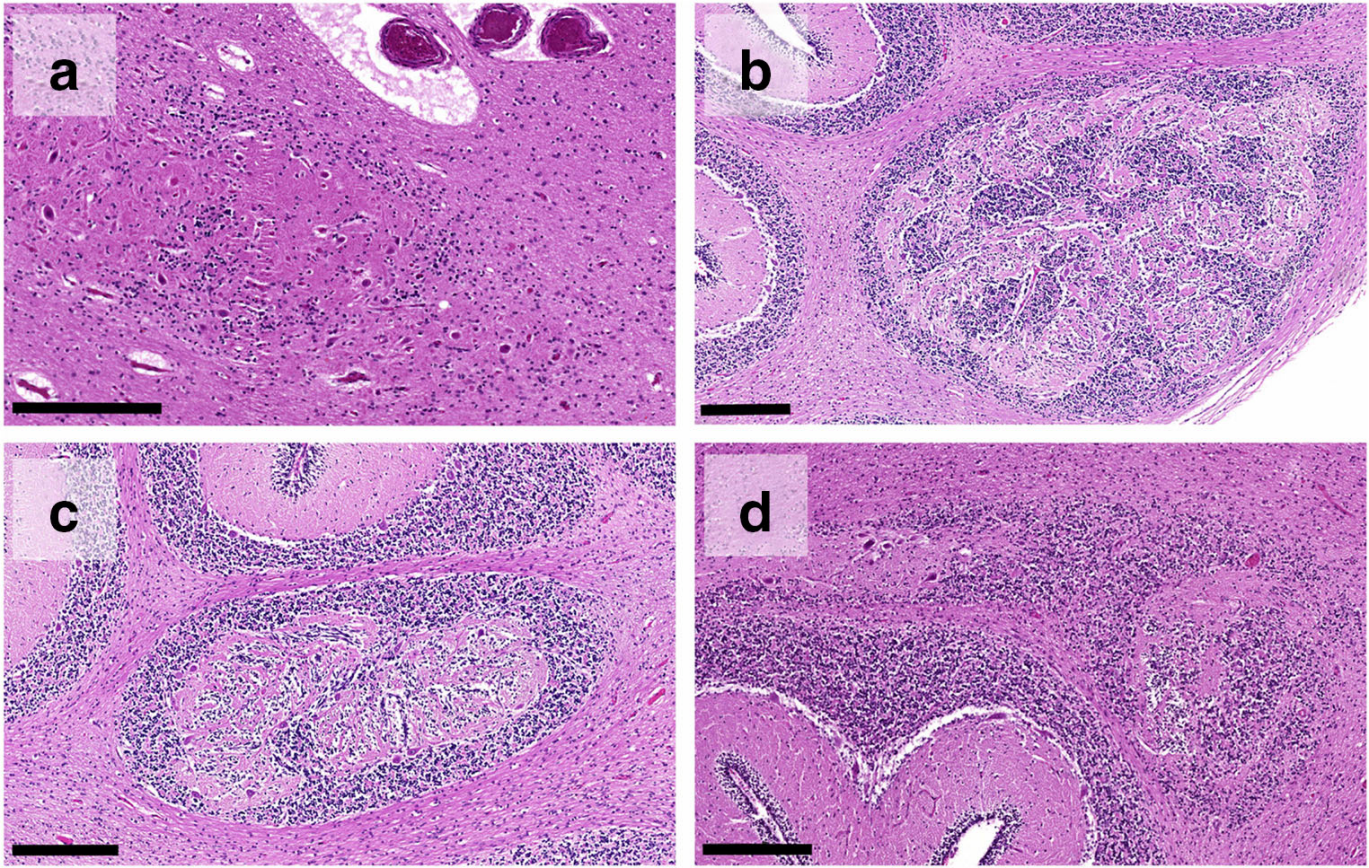
Loosely aggregated misplaced poorly organised cerebellar cell rests, heterotaxia-type in cases #1 and #4, hematoxilin and eosin stain (**a**, **d**); well-organised misplaced cerebellar cell rests composed of all physiological foliar components, heterotopia-type in cases #2 and #3, HE (**b**, **c**). Scale bar 100 μm in a–d

There were signs of brain swelling and congestion with a fresh brain weight of 678 g (normal average weight compiled from several sources around 540–560 g [19, 20]). Macroscopic examination of the brain and the dura was unremarkable. Microscopy following extensive sampling of dura and brain following an internal standard procedure [21] revealed a loose group of misplaced mature neurons with Purkinje cell-like features in the white matter of the left cerebellar hemisphere (Fig. 1a). There were no other relevant findings.

### Case #2

#### History

This 1-week-old male was the first child of a 42-year-old mother. In the 40th week of an otherwise fully unremarkable pregnancy, an emergency caesarean section had been performed following premature rupture of membranes and slowing during cardiactocograpy. After treatment of neonatal pneumonia with antibiotics, the infant was discharged 5 days after birth. Two days later, the mother nursed the baby and fell asleep. On awakening, the baby was lifeless. Resuscitation measures were without success. Investigation of the circumstance and scene of death was unremarkable. A forensic autopsy was ordered by the authorities.

#### Autopsy findings

Body weight was 3390 g (39th percentile), body length 53 cm (64th percentile), and head circumference 37 cm (93rd percentile). With slight epicanthus, broadened inner canthus, and additional 6th fingers on both hands, the baby showed external dysmorphic features. During internal examination, an ostium-secundum defect of the heart was found. There was pulmonary edema. Histology showed sparse siderophages and fresh haemorrhages in the alveoli of the lungs but no signs of inflammation. Additional cytogenetic studies revealed no numerical or structural chromosomal abnormalities. After thorough discussion, the case was assigned to SIDS category II due to non-significant developmental abnormalities and being outside the age range of category IA or IB.

#### Neuropathology findings

With a fresh brain weight of 501 g, there were signs of brain swelling and congestion (normal average weight around 360–370 g). Gross examination of the brain was inconspicuous. Microscopy was unremarkable except for a focal group of misplaced mature neurons with Purkinje cell-like features in the white matter of cerebellar vermis (Fig. 1b).

### Case #3

#### History

This 7-week-old male infant had been completely healthy until his father took him for a walk in a baby sling and finding him lifeless on returning. Resuscitation measures were unsuccessful. Investigation of circumstance and scene of death was inconspicuous. A forensic autopsy was ordered by the authorities.

#### Autopsy findings

External examination was unremarkable. Body length was 61 cm (96th percentile); body weight and head circumference had not been recorded. On internal examination, enlargement of the thymus with numerous petechial haemorrhages was noted. There was also slight enlargement of the heart but no internal malformations or signs of infection. The case was assigned to SIDS category IB.

#### Neuropathology

With a fresh brain weight of 600 g (normal average weight around 520 g), there were signs of brain swelling and congestion. Gross examination showed unilateral cerebellar hypoplasia (not shown) and microscopy revealed clumps of disorganised and misplaced cerebellar tissue in the cerebellar vermis (Fig. 1c). There were no other relevant findings.

### Case #4

#### History

This 8-week-old female infant had been in complete health prior to death when the father wearing the girl in a baby sling noticed her lifeless on returning from a walk. The father drove to the nearest hospital, but the girl was declared dead on arrival. Review of the circumstances of death was without any abnormalities or suspicious findings. A forensic autopsy was performed on behalf of the authorities.

#### Autopsy findings

External examination showed no abnormalities. Body weight was 2894 g (7th percentile), body length 47.5 cm (2nd percentile), and head circumference 34.5 cm (20th percentile). Apart from pulmonary edema, there were no significant findings. Histology showed no abnormal findings, except for a mild fatty change of the liver and sparse protein casts in the proximal tubuli of the kidneys. The case was assigned to SIDS category IB.

#### Neuropathology

Fixed brain weight was 471 g (normal value 469 g). Gross examination of the brain was without any pathologic findings. Microscopically, there were small old subdural haemorrhages considered compatible with obstetric intracranial bleedings [22–24]. Due to concerns of abusive head trauma, further extensive microscopic studies were performed but failed to reveal retinal bleedings or findings indicating diffuse or local traumatic axonal injury with immunohistochemistry for amyloid-precursor protein (APP) [21]. In the cerebellar vermis, an aggregation of disorganised and misplaced cerebellar tissue was seen (Fig. 1d), accompanied by findings compatible with olivary heterotopia in the medulla oblongata (not shown).

## Discussion

We report 4 cases of unexpected sudden death in infants ranging from 1 week to 3 months of age [3]. Since all four cases remained unexplained after complete autopsy and review of the circumstances of death, conceptually, all may be ascertained as SIDS according to the San Diego classification, or as unexplained sudden death in infancy (USDI) following the most recent suggestions from 2019 [3].

Detailed neuropathological investigation in all our four cases revealed cerebellar heterotopia of infancy (CHOI), a distinct developmental anomaly of the cerebellum (accompanied by additional olivary heterotopia in case #4). CHOI is defined as the misplaced and/or disorganised aggregation of mature or immature neuroepithelial cells in the cerebellum [25, 26] In our cases, the lesion was located near the midline in 3 cases (case #2, #3, and #4), and in one case laterally in the cerebellar hemisphere (case #1), in accordance to findings described in the literature [26]. The displaced cerebellar cell rest recapitulated all components of the physiological cerebellar folium in cases #2 and #3 (classic heterotopia according to the classification of Brun from 1917 (cit. in [26]), while the more disorganised nature and the high content in immature neuroepithelial cells in cases #1 and #4 lead us to classify these as “heterotaxia”-type (see Fig. 1). Since CHOI is nearly exclusively found in infants, it has been suggested, that it might simply disappear or regress with age [25]. On the other hand, since CHOI has so far only been found during autopsy, this circumstance might rather indicate a possible role of CHOI in the course of events leading to death. To further strengthen this suggestion, over a period of the last 20 years, we have never seen CHOI in 30 infants succumbing to abusive head trauma [21], while the 4 cases reported here were from a cohort of 18 SIDS cases over the same timespan (i.e., 22%).

One mechanism might be that CHOI is simply a morphological marker indicating more subtle, yet functionally important abnormalities of the brainstem leading to impaired protective responses. In that regard, CHOI might be a surrogate marker for more profound disturbances that are not apparent with simple light microscopy, similar to the proposed mechanism with subtle dentate gyrus abnormalities that have been shown to occur in SIDS [27, 28]. In addition, CHOI may be found in the context of more widespread migration disorders and/or in certain genetic syndromes, as in trisomies 13 or 18, emphasising a character of a proper malformation of its own [26]. The fact, that in case #2, there were external features suggestive of a possible genetic syndrome (yet evading detection by chromosomal studies), and that CHOI in case #4 was accompanied by additional olivary heterotopia, might be seen in this context.

Furthermore, a role of the cerebellum in the pathogenesis of SIDS has recently emerged, possibly by modulating appropriate responses to hypoxia/hypercapnia and/or hypotension [13]. Accordingly, a mouse model of developmental cerebellar Purkinje cell loss has been shown to lack sufficient compensatory mechanisms following experimental hypercapnia [29]. In rats with experimentally reduced numbers of cerebellar Purkinje cells, disturbed modulation of respiration has been shown, probably due to interference with the physiological inhibition on respiration by the cerebellar cortex [30]. In men, case studies have emphasised the role of the cerebellum for respiratory control [9–11, 14–16]. Patients with congenital central hypoventilation syndrome (a developmental condition with a loss of the urge to breathe during sleep—a mechanism similar to the proposed events in SIDS) show distinct features in their cerebellum with magnetic resonance imaging [31]. Similar findings have been reported in adult patients with obstructive sleep apnea [32]. In a neuropathological study of 19 SIDS cases and 12 age-matched controls, cases showed a significantly higher density in the external granular cell layer of the cerebellum [11]. The EGL begins to develop during foetal life and persists until around the end of the first postnatal year, by when the cells of the EGL have completely migrated into the internal granular cell layer of the cerebellum [33]. Therefore, a tendency of the EGL cells to persist longer might be an indicator for delayed maturation of the cerebellum in SIDS. Interestingly, both the arcuate nucleus and the inferior olivary nucleus (brainstem nuclei that share a common developmental ancestry with the EGL cells from the rhombic lip at the dorsal pontomedullary junction) have consistently shown morphological or biochemical abnormalities in SIDS [6, 34–37]. In addition, we and others have seen cases of adults with sudden unexpected death due to central apnea due to developmental anomalies or other pathological findings in the lower brainstem (Ondine´s curse; [38, 39]). In conclusion, although our study is retrospective and, therefore, purely observational, finding CHOI in four SIDS cases might serve as an encouragement for further studies on the histopathology and possible functional impairment of the cerebellum in SIDS.

Extensive histopathological studies are an important component to establish the cause of death in sudden unexpected death in infancy and are necessary to make a diagnosis of SIDS [40, 41]. It has been argued that neuropathological examinations might only rarely be of help [42]. On the other hand, the importance of detailed investigations of critical brain regions involved in arousal and/or respiratory control should be considered of utmost priority for further elucidating the mechanism behind this grave and devastating event [6, 43, 44]. Furthermore, recent studies suggest that detailed knowledge about an infant´s death might be supportive for bereaved parents [27, 28]. Accordingly, neuropathology can be of considerable help in this process.
